# The postbiotic sodium butyrate synergizes the antiproliferative effects of dexamethasone against the AGS gastric adenocarcinoma cells

**DOI:** 10.3389/fnut.2024.1372982

**Published:** 2024-03-12

**Authors:** Radwa A Eladwy, Muhammad A. Alsherbiny, Dennis Chang, Mohamed Fares, Chun-Guang Li, Deep Jyoti Bhuyan

**Affiliations:** ^1^NICM Health Research Institute, Western Sydney University, Penrith, NSW, Australia; ^2^Department of Pharmacology, Faculty of Pharmacy, Egyptian Russian University, Badr City, Egypt; ^3^Pharmacognosy Department, Faculty of Pharmacy, Cairo University, Cairo, Egypt; ^4^School of Pharmacy, The University of Sydney, Sydney, NSW, Australia; ^5^School of Science, Western Sydney University, Penrith, NSW, Australia

**Keywords:** postbiotics, gastric cancer, sodium butyrate (NaB), synergy, dexamethasone, proteomics, gut microbiome, gut microbiota

## Abstract

A growing body of literature underlines the fundamental role of gut microbiota in the occurrence, treatment, and prognosis of cancer. In particular, the activity of gut microbial metabolites (also known as postbiotics) against different cancer types has been recently reported in several studies. However, their in-depth molecular mechanisms of action and potential interactions with standard chemotherapeutic drugs remain to be fully understood. This research investigates the antiproliferative activities of postbiotics- short-chain fatty acid (SCFA) salts, specifically magnesium acetate (MgA), sodium propionate (NaP), and sodium butyrate (NaB), against the AGS gastric adenocarcinoma cells. Furthermore, the potential synergistic interactions between the most active SCFA salt-NaB and the standard drug dexamethasone (Dex) were explored using the combination index model. The molecular mechanisms of the synergy were investigated using reactive oxygen species (ROS), flow cytometry and biochemometric and liquid chromatography-mass spectrometry (LC–MS)-driven proteomics analyses. NaB exhibited the most significant inhibitory effect (*p* < 0.05) among the tested SCFA salts against the AGS gastric cancer cells. Additionally, Dex and NaB exhibited strong synergy at a 2:8 ratio (40 μg/mL Dex + 2,400 μg/mL NaB) with significantly greater inhibitory activity (*p* < 0.05) compared to the mono treatments against the AGS gastric cancer cells. MgA and NaP reduced ROS production, while NaB exhibited pro-oxidative properties. Dex displayed antioxidative effects, and the combination of Dex and NaB (2,8) demonstrated a unique pattern, potentially counteracting the pro-oxidative effects of NaB, highlighting an interaction. Dex and NaB individually and in combination (Dex:NaB 40:2400 μg/mL) induced significant changes in cell populations, suggesting a shift toward apoptosis (*p* < 0.0001). Analysis of dysregulated proteins in the AGS cells treated with the synergistic combination revealed notable downregulation of the oncogene TNS4, suggesting a potential mechanism for the observed antiproliferative effects. These findings propose the potential implementation of NaB as an adjuvant therapy with Dex. Further investigations into additional combination therapies, in-depth studies of the molecular mechanisms, and *in vivo* research will provide deeper insights into the use of these postbiotics in cancer, particularly in gastric malignancies.

## 1 Introduction

Despite the recent advances in oncology, cancer has remained an enormous global health burden, accounting for about 10 million deaths in 2020 (1). Gastric cancer (GC) is the fifth most common malignancy and the third most common cause of cancer death globally (2). GC has a poor survival rate and represents the most newly diagnosed cancer cases in Asia and South America (3). Factors that increase the risk of GC include microbial infections, such as Epstein–Barr Virus and *Helicobacter pylori*, smoking, older age, sex (males), family history, and a diet low in fruits and vegetables (3). Despite the severe side effects, chemotherapy is considered the standard in GC treatment (1).

Microbiota refers to a vast number of interacting bacteria, fungi, eukaryotic viruses, archaea, and bacteriophages coexisting with the host for potential mutual benefit (4). In the last two decades, extensive research has been done to explore the nature and therapeutic potential of gut microbiota, including its role in protection from pathogens, maintaining metabolic, endocrine, and immune functions, and modifying drug action and metabolism (5). Preclinical and clinical studies have reported the key role of human gut microbiota in different cancer types (6). Three recent reviews from our research group have underlined the key gut microbial metabolites (also known as postbiotics) with potential anticancer activity and their prospective clinical implementation after further preclinical and clinical studies (6–8). Recent reports show a strong connection between an imbalance in gut microbial communities (called dysbiosis) and the development of gastric cancer (GC). This imbalance affects not only the types of bacteria in the gut but also changes in how they function (9, 10). Specifically, certain substances produced by these microbes, such as short-chain fatty acids (SCFAs), can impact the functions and immune responses of the body (11). Changes in these substances are associated with abnormal signaling pathways in GC, highlighting the complex relationship between gut microbes and the development of tumors (11).

Gut microbial metabolites, also known as postbiotics, are a range of substances such as SCFAs, enzymes, peptides and other bioactive molecules produced in the gut. SCFAs are small monocarboxylic acids comprised mainly of acetic, propionic, and butyric acids (12). SCFAs are bioactive by-products produced by gut microbial communities, principally formed by fermentation of the undigested starch and non-starch polysaccharides in the large bowel (12). In particular, postbiotics are the outcomes of the metabolic processes of probiotics after consuming prebiotics (13–15). SCFAs are metabolized by the colonocytes and the unmetabolized fractions are transported into the portal circulation to be used as an energy source for the hepatocytes, except for acetate which is barely oxidized in the liver (16). As a result, only a small amount of SCFAs, originating from the colon, reaches the systemic circulation, so their fecal concentration has been used as a proxy of the SCFA production in the colon (5). Postbiotics have shown a broad spectrum of biological activities including anticancer and immunomodulatory functions (6, 17, 18). Several *in vitro* and *in vivo* studies have also underlined the antiproliferative effects of certain postbiotics against different cancer types, including breast, lung, prostate, colon, and stomach cancers (7). However, their molecular mechanisms of action have not been investigated adequately. Postbiotics such as butyrate, propionate, nisin, and inosine have been reported to induce apoptosis in colorectal cancer cells (6, 19).

Butyric acid has been shown to reprogram gene expression in human malignant epithelial and lymphoid cells (20). Reports have also suggested that SCFAs including butyrate, propionate, isobutyric acid, and acetic acid exhibited anti-proliferative activity against human gastric (Kato III) and colon cancer (Caco-2, DLD-1, and WirDr) cells with butyrate displaying a greater activity than the other SCFAs (21–25). These studies also highlighted that the antiproliferative activity of these SCFAs was mediated through the modulation of the cell cycle, DNA replication, recombination, and repair and apoptosis. Sodium butyrate (the sodium salt form of butyric acid) was also found to induce death-associated protein kinase (DAPK) expression, which led to apoptosis by reducing the FAK protein level in the AGS and MKN45 human GC cells (26). Another study demonstrated that the SCFA-propionate enhanced the cytotoxic effect of cisplatin by regulating GPR41 signaling pathways in the HepG2 liver cancer cells (27).

The current work aimed to gain a comprehensive understanding of the antiproliferative activities of SCFA (acetate, propionate, and butyrate) salts, and explore their interactions with dexamethasone (Dex) against GC cells *in vitro*. Apoptotic profiles of the cells after treatment with SCAFs, Dex, and their most effective combination were evaluated using flow cytometry. Furthermore, a shotgun proteomics study was carried out to uncover the proteomic-level insights into the mechanisms of cytotoxicity and synergy of SCFA, along with a focus on the most notable synergistic combination.

## 2 Materials and methods

### 2.1 Chemicals and drug preparation

All the solvents used in the study are of analytical quality and were procured from Sigma Aldrich (Castle Hill, NSW, Australia). Magnesium acetate (MgA), sodium propionate (NaP), sodium butyrate (NaB), dexamethasone (Dex) and doxorubicin (Dox) were also purchased from Sigma Aldrich (Castle Hill, NSW, Australia). Furthermore, all reagents were prepared according to the standard methods and protocols provided with the assay kits.

### 2.2 Cell culture

AGS (CRL-1739, ATCC) and HS738.St/Int cell lines (CRL-7869, ATCC) were purchased from the American Type Culture Collection (ATCC, United States). AGS cells were grown in the ATCC-formulated F-12 K medium (Kaighn’s Modification of Ham’s F-12 Medium) containing 2 mM L-glutamine and 1500 mg/L sodium bicarbonate with 10% fetal bovine serum (Bio-Strategy PTY, Campbellfield, VIC, Australia), and supplemented with 1% penicillin and streptomycin (Sigma-Aldrich, Castle Hill, NSW, Australia). The HS738.St/Int cells were grown in the ATCC-formulated DMEM (Dulbecco’s Modified Eagle Medium) comprised of 4.5 g/L glucose, L-glutamine, and sodium pyruvate supplemented with 10% fetal bovine serum (Bio-Strategy PTY Campbellfield, VIC, Australia), and supplemented with 1% penicillin and streptomycin (Sigma Aldrich, Castle Hill, NSW, Australia). These cells were maintained at 37°C in a 5% controlled CO_2_ atmosphere and cell maintenance was performed every 48–72 h, the time necessary for cells to achieve confluent monolayers.

The murine RAW264.7 macrophage cells were cultured in DMEM (Lonza Australia, Mount Waverley, VIC, Australia) supplemented with 5% FBS (Bio-Strategy PTY Campbellfield, VIC, Australia), and supplemented with 1% penicillin and streptomycin (Sigma-Aldrich, Castle Hill, NSW, Australia) at 37°C in a humidified incubator with 5% CO_2_.

### 2.3 Cell viability assays

The cell viability of the AGS cells after treatment with different concentrations of the three postbiotics (MgA, NaP, NaB) and Dex was determined using the Alamar blue assay as per the method described earlier (28, 29). Briefly, 100 μL of cells were cultured in 96 well-plates at a seeding density of 10^5^ cells/mL. After 24 h, the cells were treated with the compounds and incubated for 72 h. A positive control using Dox at a concentration of 1 μM was included, and a negative control with 0.1% DMSO was added to every plate. At the end of the incubation period, the culture media were removed, and 100 μL of a 0.1 mg/mL Alamar blue solution (resazurin, prepared as a stock solution at 1 mg/mL in freshly made PBS followed by a 1:10 dilution with serum-free media) was added to each well. The fluorescence levels were assessed with a microplate spectrophotometer (BMG CLARIOstar, Mornington, VIC, Australia) using an excitation wavelength of 555 nm and measuring emission at 595 nm. The compounds were tested in triplicate, with the negative control taken as 100% cell viability.

### 2.4 Synergy analysis

Dex was combined with the most active postbiotic in nine different ratios (1:9, 2:8, 3:7, 4:6, 5:5, 6:4, 7:3, 8:2, and 9:1, v/v) for combination index (CI) analyses. The potential interactions between Dex and the most active postbiotic were analyzed using the CI model and the CompuSyn version 2.0 (Biosoft, CA, United States) was used for the CI calculations based on the median-effect equation, which was derived from mass action law (28). In the current study, the nine pairwise combinations of Dex and the postbiotic were studied with a six-point dose–response curve using the CI model.

### 2.5 ROS production analysis

The effect of the most active gut metabolites and their most synergistic combinations on the oxidative stress of the cancer cells was studied as per our recently reported protocol using the H2DCFDA (2′,7′-dichlorofluorescein diacetate) cellular reactive oxygen species (ROS) Detection Assay Kit (#ab113851; Abcam, Melbourne, VIC, Australia) (19, 29). Briefly, AGS cells (2.5 × 10^5^ cells/mL) were cultured in a 96-well plate, adhered overnight, and treated with 20 μM H2DCFDA for 45 min to assess ROS levels. The dye solution was removed, and cells were washed with 1x buffer. Next, the cells were treated with 750, 1,500 and 3,000 μg/mL of SCFAs, 50, 100 and 200 μg/mL of Dex, 0.54 μg/mL (1 μM) of Dox, and 250 μM of tert-Butyl hydroperoxide (TBHP) and incubated at 37°C for 4 h. Finally, the plate was immediately read at Ex/Em = 485/535 nm using a microplate spectrophotometer (BMG CLARIOstar, VIC, Australia). The fold increase in ROS production was determined relative to the untreated control (cells treated with the supplement buffer according to the manufacturer’s protocol).

### 2.6 Anti-inflammatory assay

Nitric oxide (NO) production in the RAW264.7 macrophage cells, stimulated with lipopolysaccharides (LPS), was assessed by measuring total nitrite content using Griess reagents (a mixture of an equal amount of 1% sulphanilamide in 5% phosphoric acid and 0.1% N-1-(naphthyl)ethylenediamine dihydrochloride). The cells were seeded at a density of 0.85 × 10^6^/mL in a 96-well culture plate, incubated for 45 h, treated with different concentrations of Dex, the most active postbiotic and their combination and then stimulated with 50 ng/mL of LPS for an additional 16 h. Cell supernatant was collected, mixed with Griess reagents, and absorbance was measured at 540 nm using a microplate spectrophotometer (BMG CLARIOstar, Mornington, VIC, Australia).

### 2.7 Flow cytometry

The impact of the most potent postbiotic and its most synergistic combination with Dex on the apoptosis profiles of the AGS adenocarcinoma cells was studied using an annexin V and 7-AAD-based kit (#ab214663, Abcam, Melbourne, VIC, Australia) (19, 29). The AGS cells were cultured in T75 cell culture flasks with an initial density of 1 × 10^6^ cells per 10 mL at 37°C in the presence of 5% CO_2_ for 24 h. The following day, the cell culture media was removed from each flask and replaced with fresh FBS-containing media, and the cultured flasks were then treated with the highest concentration of the most active postbiotic (3,000 μg/mL), 200 μg/mL of Dex, 0.54 μg/mL (1 μM) of Dox (used as a positive control). Serum-containing medium was used as the untreated control. The flasks were then incubated at 37°C with 5% CO_2_ for an additional 24 h.

The apoptotic profiles of the AGS cells after 24 h were studied using the Abcam apoptosis detection kit following the manufacturer’s instructions. Briefly, the cell culture media from each flask was collected. Subsequently, trypsin (0.25% *w/v*) was applied to the flasks for 3 min at 37°C. The trypsin reaction was neutralized with an equal volume of 10% FBS serum-containing media, and the cells were combined with the previously collected media. The cell pellets were obtained by centrifuging at 500 × *g* for 5 min at room temperature (RT). This procedure was repeated by suspending the cell pellets in 1 mL of PBS each time. The collected cell pellets from each treatment were immediately suspended in 500 μL of 1x binding buffer and gently mixed by pipetting. Annexin V-*CF* blue (5 μL) and 7- AAD (5 μL) staining solutions were added to 100 μL of cell suspension. The stained cells were incubated for 15 min in the dark at RT, after which 400 μL of a 1x assay buffer was added to each cell suspension. Subsequently, the cells were examined using a flow cytometer (Novocyte 3,000, ACEA Biosciences Inc., CA, United States), and data analysis and processing were performed using NovoExpress software (version 1.5.0, ACEA Biosciences Inc., CA, United States). In the initial step, the cells were gated on forward and side scatter modes to exclude cell aggregates and debris near the origin. Following that, the cells were gated on dot plots, where Annexin V-*CF* in Pacific Blue was plotted against 7-AAD fluorescence in PerCP. Quadrants were positioned relative to the untreated control, indicating live cells (+Annexin V and − 7-AAD) appearing in the lower-left quadrant, early apoptotic cells (+Annexin V and − 7-AAD) in the lower-right quadrant, late apoptotic cells (+Annexin V and + 7-AAD) in the upper-right quadrant, and necrotic cells (−Annexin V and + 7-AAD) in the upper-left quadrant. For statistical analyses and visualization, the percentage data of cells in each quadrant after different treatments (*n* = 3) were exported to GraphPad Prism software (version 9.0, San Diego, CA, United States).

### 2.8 Liquid chromatography-mass spectrometry (LC–MS)-driven bottom-up proteomics analysis

#### 2.8.1 Cell culture, treatment, and protein extraction

The AGS adenocarcinoma cells were initially placed in T75 flasks at a concentration of 1.0 × 10^5^ cells/mL and were allowed to incubate overnight at 37°C in the presence of 5% CO_2_. After removing the media, it was replaced with fresh F-12 K medium supplemented with 10% FBS, and the cultured flasks were treated, as well as specific doses of the most active postbiotic, Dex, and their combinations. Treatments were done in triplicate and incubated for 24 h under the same conditions as before. Following the incubation, each flask of cells was subjected to 0.25% w/v trypsin treatment for 3 min at 37°C, and the cell culture media were collected. To neutralize the trypsin, an equal volume of media containing F-12 K medium (containing 10% FBS) was added before mixing with the previously collected media. The cells were then spun in a centrifuge at 500× *g* for 5 min at RT. The cell pellets were subsequently washed twice with ice-cold PBS and spun again at 500× *g* for 5 min. These cell pellets were then suspended in a lysis buffer that included 1 μL of universal nuclease and a Halt™ Protease and Phosphatase Inhibitor Cocktail (Thermo Fisher Scientific, Sydney, NSW, Australia) that was fully compatible with mass spectrometry (MS). The cells were gently pipetted around 10–15 times to reduce the viscosity of the sample, after which it was placed on ice for 20 min. The lysate was then centrifuged at 14,000 rpm for 20 min at 4°C, and the resulting liquid was collected.

#### 2.8.2 Protein quantification

The Pierce™ Rapid Gold BCA Protein Assay Kit (#A53226, Thermo Fisher Scientific, Sydney, NSW, Australia) was used to determine the protein concentration of the cell lysate in triplicate, using a bovine serum albumin (BSA) standard, following the manufacturer’s protocol (19). In brief, 1 μL of each sample replicate was diluted 1:20 in water, along with 20 μL of each standard, and then placed in a 96-well plate with 200 μL of working reagent in each well. Samples were diluted until they were within the working range of 20–2,000 μg/mL. The plate was thoroughly mixed on a plate shaker for 30 s, incubated at RT for 5 min, and then the absorbance was measured within 20 min at 480 nm using a microplate spectrophotometer (BMG CLARIOstar, Melbourne, VIC, Australia). The blank absorbance was subtracted from all other readings of standards and samples, and the sample concentration was determined using the established BSA standard calibration curve. The samples were then stored at −80°C for further analysis.

#### 2.8.3 Peptides preparation and clean-up

The protein samples (100 μg) were subjected to chemical and enzymatic sample processing using the EasyPep^™^ Mini MS Sample Prep Kit following the manufacturer’s instructions (Thermo Fisher Scientific, Sydney, NSW, Australia) and as reported in literature (19). Briefly, the sample volume was then adjusted to 100 μL using a lysis buffer in a microcentrifuge tube. Subsequently, the reduction and alkylation solutions (50 μL each) were introduced, gently mixed, and incubated at 95°C with a heat block for 10 min. The samples were allowed to cool to RT, after which 50 μL of the reconstituted trypsin/lys-C protease mixture was added to each sample and incubated with shaking at 37°C for 3 h. Following incubation, 50 μL of a digestion stop solution was gently mixed into the samples. Peptide clean-up columns were employed to eliminate hydrophilic and hydrophobic impurities. The resulting clean peptide samples were dehydrated using a vacuum centrifuge and reconstituted in 100 μL of a 0.1% formic acid solution in water for LC–MS analysis. Subsequently, these samples were carefully transferred to maximum recovery sample vials (Waters Corp., Milford, MA, United States).

#### 2.8.4 Label-free bottom-up quantification proteomics analysis via nano-ultra-high-performance liquid chromatography coupled with quadruple time-of-flight mass spectrometry (Nano-UPLC-qTOF-MS)

The tryptic peptides were analyzed as reported in literature using a nanoACQUITY UPLC system manufactured by Waters Corp. (Milford, MA, United States) paired with a Synapt G2-S high-definition mass spectrometer (HDMS, Waters Corp., Manchester, United Kingdom) (19, 28). This mass spectrometer operated in positive electron spray ion mode (ESI^+^) equipped with a hybrid quadrupole time of flight (qTOF) analyzer. To ensure precise mass accuracy, a Waters NanoLockSpray Exact Mass Ionization Source was employed.

Exactly 100 fg/mL solution of Glu-fibrinopeptide B (GFP), with a lock mass of m/z 785.84.26 and dissolved in 50% aqueous acetonitrile containing 0.1% formic acid, was infused into the lock spray solution. This solution was infused at a rate of 0.5 μL/min and was calibrated using a sodium iodide solution. For peptide separation, a nanoEase M/Z BEH C18 column (1.7 μm, 130 Å, 75 μm × 100 mm; Waters Corp., Milford, MA, United States) was employed, operating at 40°C. This column was coupled with a nanoEase M/Z Symmetry C18 Trap Column (100 Å, 5 μm, 180 μm × 20 mm; Waters Corp.). Milli-Q water and acetonitrile containing 0.1% formic acid were used as mobile phases A and B, respectively, and were of LCMS grade from Merck (Macquarie Park, NSW, Australia). The injection volume was 1 μL, and the flow rate was set to 300 nL/min over a 50-min gradient. Initially, samples were injected into the trapping column at 5 μL/min using 99% mobile phase A for 3 min before elution into the analytical column. The gradient transitioned from 1% of mobile phase B to 85% B over 50 min, with specific points at 10% B at 2 min, 40% B at 40 min, and 85% B at 42 min. All samples were maintained at 4°C and were duplicated for injection. Operating conditions for the mass spectrometer included a source block temperature of 80°C and a capillary voltage of 3 kV. Ions were acquired within the m/z range of 50 to 2000, with a scanning time of 0.5 s. The sample cone voltage and source offset were set at 30 V, and nanoflow gas was maintained at 0.3 Bar, while purge gas flowed at 20 L/h, and cone gas flow at 20 L/h. The acquisition method utilized a data-independent acquisition (DIA) through MSE multiplex mode, incorporating a T-wave collision-induced dissociation cell filled with argon gas. Data acquisition and analysis were conducted using MassLynx Mass Spectrometry Software from Waters Corp. (Milford, MA, United States).

#### 2.8.5 Data processing and availability

Progenesis QI software (Milford, MA, United States) was used to import and further analyze the data obtained from MassLynx. The software selected the alignment references automatically from quality control (QC) samples. Peptides were then matched to entries in the UniProt human proteome database using the ion accounting method, with a maximum protein mass of 250 kDa. The ion matching criteria for relative quantification were established as follows: we required either one fragment per peptide or one peptide per protein, in addition to three fragments per protein. This was achieved through the implementation of the Hi-N method with a sample size of three (*n* = 3). Auto peptide and fragment tolerance, and less than 4% FDR, were set as search tolerance parameters. Peptides with an absolute mass error greater than 20 ppm or those with a single charge were filtered out. For assessing cytotoxic potential, pairwise comparisons of the identified proteins in the treated groups were conducted against the negative control group. To study the potential synergistic mechanisms, we contrasted samples treated with the most active synergistic combinations with monotherapies. In the experimental designs, we considered proteins with analysis of variance (ANOVA)-derived *p*-values of ≤0.05 and q-values ≤0.05, along with an absolute fold change of at least 2, as significant. These significant proteins were then subjected to further pathway analyses. Pathway analyses were performed using various tools, including STRING (30), Reactome (31), g:Profiler (32), and IMPaLA (33) (supporting information), to identify pathways responsible for the observed synergistic effects against the AGS adenocarcinoma cells. The raw and processed data have been deposited in the ProteomeXchange Consortium via the PRoteomics IDEntifications (PRIDE) repository with the dataset identifiers (34) PXD048617.

### 2.9 Statistical analysis

Data were collected and managed using MS Office - Excel and GraphPad Prism for both statistical analyses and visualization. Data collection and analyses were carried out in triplicate, and the outcomes were presented as the mean ± standard deviation. Statistical significance between the mean values was determined at *p* < 0.05 employing a two-way ANOVA. To perform multiple comparisons, Tukey and Dunnett’s tests were utilized within the GraphPad Prism software. Furthermore, the IC_50_ value (representing the concentration of a drug required to achieve a 50% cell growth inhibition) was computed using GraphPad Prism software. Nonlinear regression and determination of IC_50_ values were carried out using GraphPad Prism 9.0.

## 3 Results and discussion

### 3.1 Antiproliferative activity of gut microbial metabolites and standard drug Dex

Three SCFA salts, namely MgA, NaP and NaB at different concentrations, were investigated for their anti-proliferative activity against the AGS adenocarcinoma cells following a 72-h treatment using the Alamar Blue assay. All three SCFA salts exhibited significant anti-proliferative activity against the AGS cells, with NaB showing the most significant dose-dependent activity (93.75–3,000 μg/mL) followed by NaP and MgA (Table 1). The standard anti-inflammatory and chemotherapeutic drug Dex also displayed dose-dependent activity (6.25–200 μg/mL) against the AGS cells (Table 1).

**Table 1:** Cell growth inhibition (%) against the AGS gastric adenocarcinoma and cell viability (%) of the Hs 738.St/Int normal intestine cell lines at different concentrations of sodium butyrate (NaB), sodium propionate (NaP), magnesium acetate (MgA), and dexamethasone (Dex) for 72 h using the Alamar Blue assay (*n* = 3).

Conc. μg/mL	Cell growth inhibition (%)			Cell viability (%)	Conc. μg/mL	Cell growth inhibition (%)	Cell viability (%)
	AGS			HS738.St/Int		AGS	HS738.St/Int
	NaB	NaP	MgA	NaB		Dex	
3,000	100.36 ± 1.23 ^a,x^	95.15 ± 1.99 ^a,x^	81.47 ± 20.28 ^a,x^	106.91 ± 9.25 ^a^	200	80.46 ± 8.08 ^a^	106.86 ± 2.58 ^a^
1,500	99.62 ± 0.84 ^a,x^	81.51 ± 4.79 ^a,x^	54.12 ± 35.77 ^a,y^	115.50 ± 9.41 ^a^	100	39.38 ± 24.51 ^b^	115.48 ± 2.92 ^a^
750	98.12 ± 1.43 ^a,x^	53.66 ± 18.87 ^b,y^	18.20 ± 42.96 ^b,z^	117.90 ± 1.45 ^a^	50	–	119.01 ± 3.87 ^a^
375	91.69 ± 5.41 ^a,x^	36.35 ± 37.01 ^b,y^	18.37 ± 43.42 ^b,y^	113.89 ± 7.33 ^a^	25	–	120.05 ± 5.19 ^a^
187.5	67.38 ± 12.03 ^a,x^	16.26 ± 40.34 ^b,y^	30.41 ± 53.75 ^b,y^	117.84 ± 2.08 ^a^	12.5	–	121.84 ± 7.95 ^a^
93.75	41.88 ± 28.47 ^b,x^	20.66 ± 43.49 ^b,x^	36.05 ± 57.01 ^b,x^	116.11 ± 2.80 ^a^	6.25	–	118.66 ± 11.95 ^a^
**IC**_**50**_	**193.97 ± 5.55**	**736.87 ± 157.56**	**1033.03 ± 694.18**	**NA**		**86.60 ± 11.85**	**NA**

NA indicates ‘not applicable’ as all tested concentrations increased the viability of the HS738.St/Int cell line. ^a,b^The different superscript values in the same column for each cell line indicate statistically significant difference (*p* < 0.05) compared to the highest concentration (3,000 μg/mL). ^x,y,z^The different superscript values in the same row for the AGS gastric adenocarcinoma cell line indicate statistically significant difference (*p* < 0.05) between the treatment groups.

The antiproliferative effect of postbiotics has been extensively investigated in different types of cancer including breast and colon cancers; however, only a few studies have explored the antiproliferative action of postbiotics on GC (8, 35–37). In a previous study, NaB at 4.0 mM was found to inhibit the growth of the AGS cells by 81.54% using the colorimetric MTS assay (26). Other studies found that the inhibitory rate of NaB on AGS cells could reach 59.19% at an intervention concentration of 10 mmol/L using the MTT assay (38). Another *in vitro* study from our research group showed that 3,000 μg/mL (27.25 mM) NaB treatment showed 81.73% growth inhibition of the MCF7 cells using Alamar Blue assay (19). In the current study, 3,000 μg/mL (27.25 mM) and 1,500 μg/mL (18.17 mM) of NaB exhibited AGS anti-proliferative activity of 100.36 ± 1.23% and 99.62 ± 0.84%, respectively using Alamar blue assay with the IC_50_ value of 193.97 ± 5.55 μg/mL (1.76 mM; Table 1).

NaP displayed a growth inhibition value of 95.15 ± 1.99% at 3000 μg/mL, with an IC_50_ value of 736.87 ± 157.56 μg/mL (7.67 mM) against the AGS cells (Table 1). While the cell inhibition rates were not as high as those observed with NaB, this investigation stands as one of the first reports of the antiproliferative effects of NaP against the AGS gastric adenocarcinoma cells. A previous investigation examined the impact of NaP on the MCF7 and MDA-MB-231 breast cancer cells, revealing a significant decrease in the viability of both cell lines when exposed to 0.5–20 mM NaP over 48 h in a dose-dependent manner (39). In the present study, NaP emerged as the second most effective postbiotic in inhibiting the growth of AGS cells in a dose-dependent manner. Specifically, at concentrations of 3,000 and 1,500 μg/mL, NaP induced mean cell growth inhibition of 95.15 and 81.51%, respectively.

MgA demonstrated growth inhibition value of 81.47 ± 20.28% at 3000 μg/mL (21.07 mM) and 54.12% at 1500 μg/mL (10.53 mM) against the AGS cells, with an IC_50_ value of 1033.03 ± 694.18 μg/mL (7.25 mM; Table 1). Few *in vitro* studies have previously investigated the cytotoxicity and cell proliferation inhibition against the AGS cells demonstrating 50% inhibition of AGS cells at 12.5 mM (40) using Quick cell proliferation assay. Differences in outcomes between the current and the previous studies may arise from the distinct nature of acetate salts, which could exhibit variations in pharmacokinetic and pharmacodynamic activities as well as the differences in the assays implemented.

The highest concentration of Dex (200 μg/mL) resulted in substantial growth inhibition of 80.46% in the AGS cells in a dose-dependent manner (Table 1). At 100 μg/mL, 39.38% cell inhibition was observed with lower concentrations displaying no activity. The IC_50_ of Dex in AGS cells was determined to be 86.60 ± 11.85 μg/mL. The current study, to the best of our knowledge, is one of the first reports to demonstrate the antiproliferative effects of Dex against GC cells.

Testing anticancer drugs on normal cell lines is crucial to assess their selectivity and potential side effects, providing critical insights into the therapeutic index and safety profile of these drugs in order to minimize harm to healthy tissues during treatment (41). The most active SCFA NaB, and the chemotherapeutic drug Dex were tested against the Hs 738.St/Int human normal Intestine cells using the Alamar blue assay (Table 1). NaB at the highest tested concentrations of 3,000 μg/mL, and Dex at 200 μg/mL displayed cell viability values of 106.91 ± 9.25% and 106.86 ± 2.58%, respectively in the Hs 738.St/Int human normal intestine cells. This is presumably because SCFAs are the primary energy source for intestinal epithelial cells in the gut (42), allowing NaB to induce the proliferation of normal Hs 738.St/Int human normal intestine cells in our study. These results demonstrate the favorable safety profile of NaB and Dex against the healthy cells, indicating their potential for use in future therapies.

### 3.2 The synergy of sodium butyrate with dexamethasone against the AGS cells

The potential synergistic interactions between Dex with NaB were evaluated using the CI model as per our previous study (28). The CI model quantifies the potential interactions between drug–drug combinations into three categories: (a) synergistic effect: CI value <1, (b) additive effect: CI = 1, and (c) antagonistic effect: CI value >1. Nine different ratios of Dex and NaB (1:9, 2:8, 3:7, 4:6, 5:5, 6:4, 7:3, 8:2 and 9:1; Table 2) were evaluated against the AGS cells and strong synergistic interactions were observed between Dex and NaB at a 2:8 (40 μg/mL Dex + 2,400 μg/mL NaB) ratio with CI values <1 compared to the mono treatments of Dex and NaB. The synergistic ratio was also evaluated for its impact on the cell viability (%) of the Hs 738.St/Int normal intestine cell line (Table 2).

**Table 2:** Drug interaction analysis of Dex and NaB combinations in the AGS gastric adenocarcinoma cells.

Combinations Dex:NaB	CI values at			
	IC_50_	IC_75_	IC_90_	IC_95_
1:9	**0.92**	**0.97**	1.02	1.05
2:8	**0.86**	**0.83**	**0.81**	**0.79**
3:7	1.91	1.70	1.52	1.41
4:6	2.37	2.12	1.89	1.75
5:5	2.42	2.13	1.88	1.72
6:4	1.96	1.79	1.63	1.53
7:3	1.15	1.14	1.13	1.12
8:2	1.53	1.40	1.28	1.21
9:1	1.33	1.26	1.19	1.14

CI, combination index; IC, inhibitory concentration. The bold numbers (CI values < 1) indicate synergistic interactions between Dex and NaB.

Following the synergy study, cell growth inhibition and cell viability of the most active combination (2:8) were evaluated in the AGS cells and the Hs 738.St/Int normal intestine cells, respectively. The synergistic combination at 305–2440 μg/mL demonstrated 80.32–103.78% cell growth inhibition of the AGS gastric adenocarcinoma cells (Table 3). All tested concentrations of the 2:8 combination improved the cell viability of the normal Hs 738.St/Int intestine cells, indicating a good safety profile (Table 3).

**Table 3:** cell growth inhibition (%) against AGS gastric adenocarcinoma and cell viability (%) of Hs 738.St/Int normal intestine cell lines at different concentrations of Dex:NaB (2:8).

Conc. (μg/mL) Dex:NaB (2:8)	Cell growth inhibition (%)	Cell viability (%)
	AGS	HS738.St/Int
2,440	103.78 ± 3.37 ^a^	109.41 ± 5.41 ^a^
1,220	102.05 ± 1.96 ^a^	111.40 ± 3.03 ^a^
610	100.03 ± 4.44 ^b^	110.50 ± 4.30 ^a^
305	80.32 ± 10.50 ^b^	101.06 ± 16.08 ^a^
152.5	36.49 ± 27.10 ^c^	118.22 ± 3.87 ^a^
76.25	-	104.95 ± 6.09 ^a^

^a,b,c^The different superscript values in the same column for each cell line indicate statistically significant difference (*p* < 0.05) compared to the highest concentration (2,440 μg/mL).

### 3.3 ROS production in the AGS cells after treatment with different concentrations of NaB, Nap, MgA. Dex and Dex:Nab (2:8)

Oxidative stress is a hallmark of carcinogenesis. Reactive oxygen species (ROS) function as a significant biomarker in cancer and play a crucial role not only in the initiation of cancer but also in its progression and metastasis (43). Consequently, inhibiting the production of ROS by cancer cells is often regarded as a valuable approach for the development of novel anticancer drugs. Our investigation is focused on examining the impact of the most potent postbiotics and their most synergistic combination on the oxidative stress levels within AGS cancer cells.

**Figure 1:**
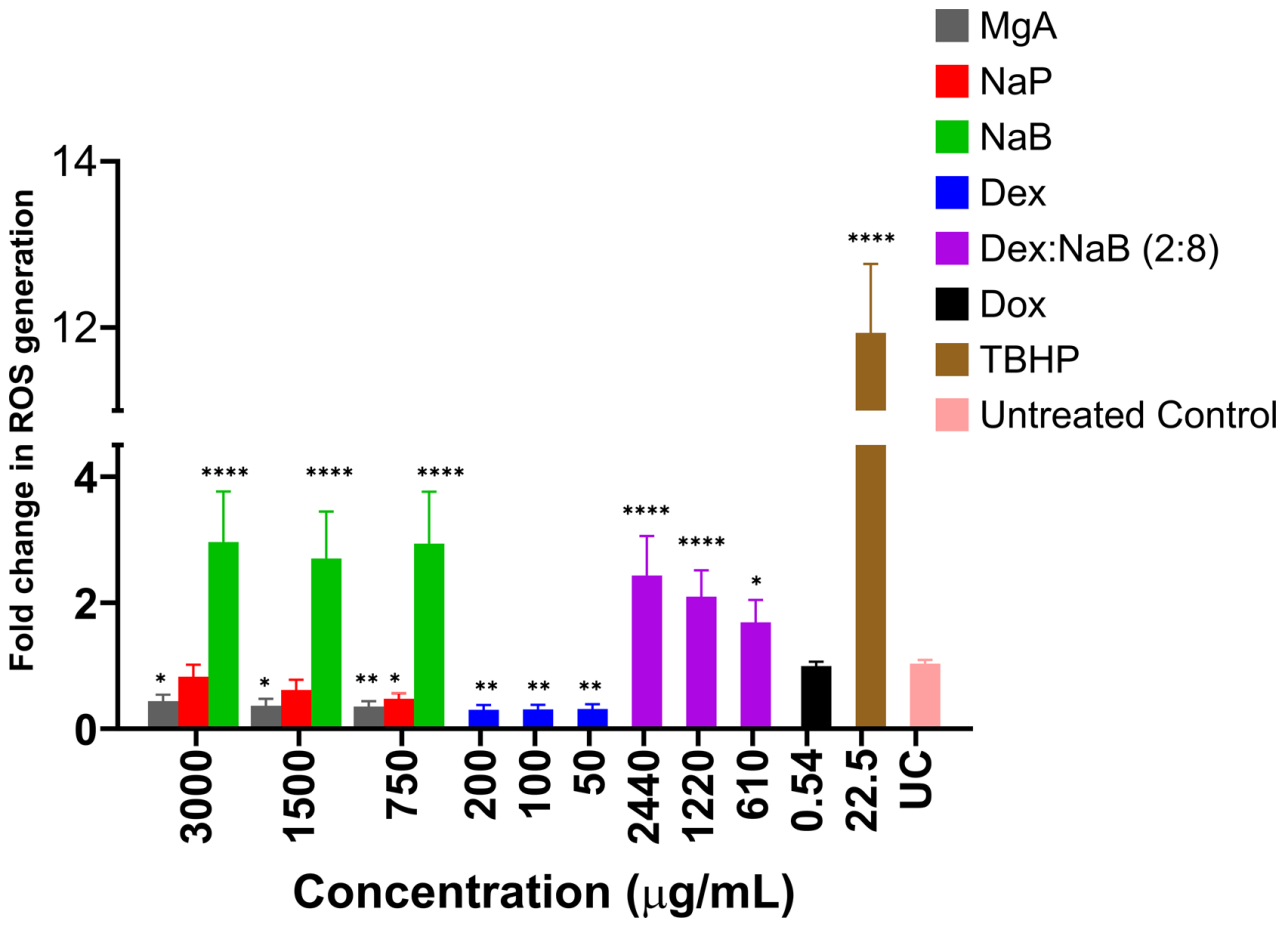
The impact of various concentrations of MgA, NaP, and NaB (3,000 μg/mL, 1,500 μg/mL, and 750 μg/mL), Dex (200 μg/mL, 100 μg/mL, and 50 μg/mL) and Dex:NaB (40:2400 μg/mL, 20:1200 μg/mL, and 10:600 μg/mL) on the production of reactive oxygen species (ROS) in the AGS gastric adenocarcinoma cells. Additionally, Dox (0.54 μg/mL or 1 μM) and tert-Butyl hydroperoxide (TBHP; 22.5 μg/mL or 250 μM) were included for comparative purposes. The values are expressed as mean ± SD. *indicates value of *p*: 0.01 < value of *p* <0.05, **indicates value of *p* ≤0.01, ****indicates *p* < 0.0001 compared to untreated control.

MgA and NaP reduced ROS production in the AGS cells at all tested concentrations (3,000 μg/mL, 1,500 μg/mL, and 750 μg/mL; Figure 1). This is in contrast with previous studies which showed that MgA and NaP induced ROS production in Caco-2 colorectal cancer cells and mouse glomerular mesangialn cells (43, 44). At all tested concentrations (3,000 μg/mL, 1,500 μg/mL, and 750 μg/mL), NaB significantly enhanced ROS production compared to the negative control (*p* < 0.0001) suggesting that NaB may have pro-oxidative properties in the AGS cells (Figure 1). Previously, it was reported NaB increased levels of ROS after 48 h at 1, 5, and 10 mM, triggering the onset of apoptosis in the MCF7 breast cancer cells (19, 45).

Treatment with Dex, (200 μg/mL, 100 μg/mL, and 50 μg/mL), displayed a significant reduction in ROS production compared to the untreated control (*p* ≤ 0.01). The lower fold change values indicated the potential antioxidative or ROS-suppressing effects of Dex in the AGS cells (Figure 1). The combination treatment showed a unique pattern of ROS modulation, representing a potential interaction between NaB and Dex. The combination demonstrated a substantial fold increase in ROS production compared to Dex alone (*p* < 0.05), but a decrease compared to NaB alone. This suggested that Dex in the synergistic combination potentially counteracted some of the pro-oxidative effects of NaB observed in isolation. The observed combination effect indicates that Dex, known for its anti-inflammatory properties, might counterbalance or mitigate the pro-oxidative impact of NaB. The ability of the combination to moderate ROS production could have implications for oxidative stress regulation, potentially highlighting a synergistic or protective interaction between NaB and Dex.

### 3.4 Anti-inflammatory activity of NaB, Dex and the most active combination Dex:Nab (2:8)

**Figure 2:**
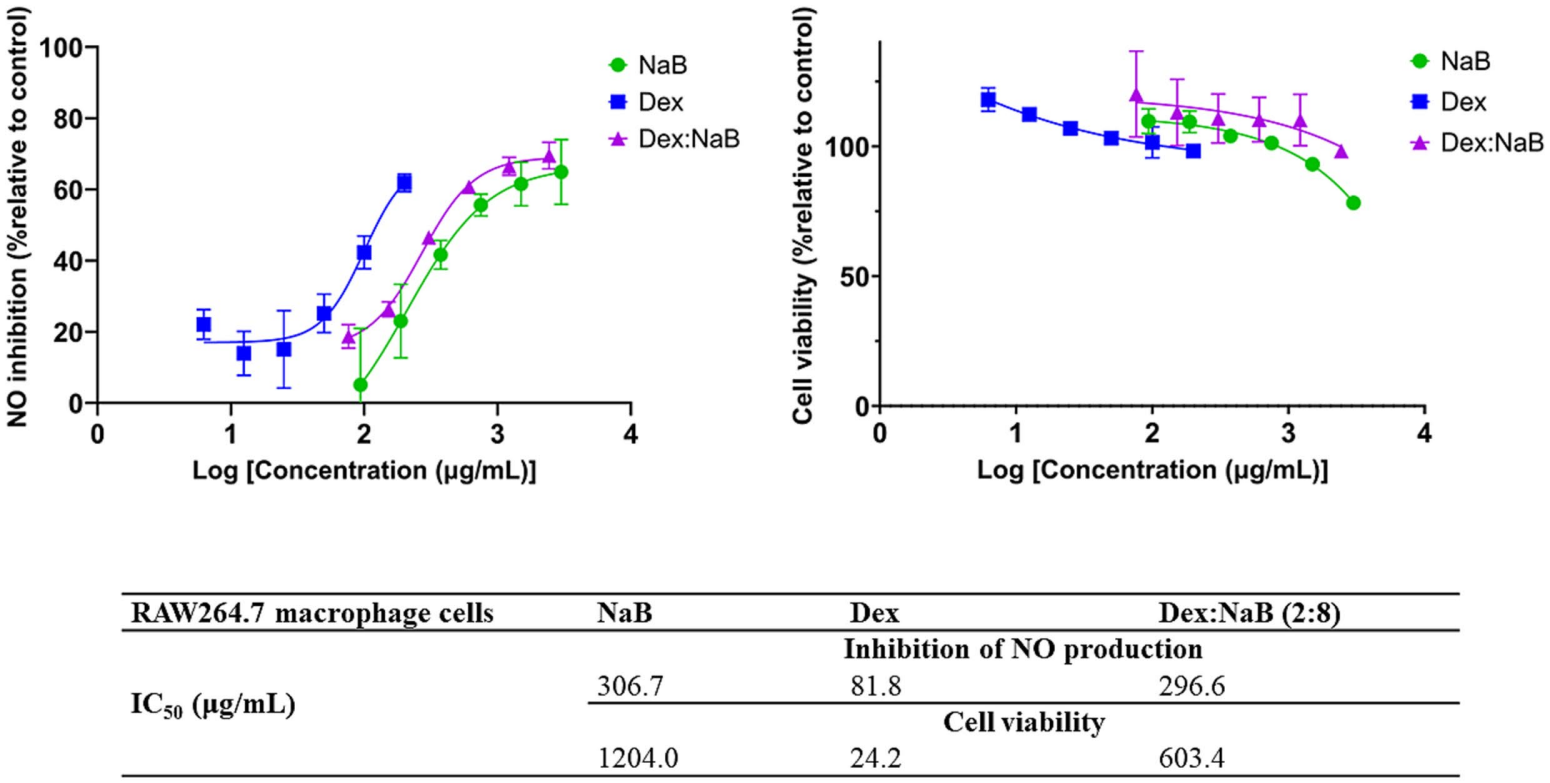
The inhibitory effect of different concentrations of NaB, Dex and Dex:NaB (2:8) on NO production and cell viability of the RAW264.7 macrophage cells (*n* = 3).

The anti-inflammatory activity of Dex, NaB, and their most synergistic combination (2:8) was measured by their ability to inhibit the production of NO in RAW264.7 macrophage cells stimulated with LPS. NaB, Dex, and their combination dose-dependently inhibited NO production, as shown in Figure 2. The standard anti-inflammatory drug Dex showed the greatest inhibition of NO production (IC_50_ = 81.8 μg/mL) in this study, as predicted. Interestingly, both NaB alone and its synergistic combination with Dex exhibited potent anti-inflammatory effects with IC_50_ values of 306.7 μg/mL 296.6 μg/mL, respectively. In the Alamar Blue assay, Dex was found to be more cytotoxic to RAW264.7 macrophage cells (IC_50_ = 24.2 μg/mL), followed by the combination Dex:NaB (IC_50_ = 603.4 μg/mL) and NaB (IC_50_ = 1204.0 μg/mL; Figure 2). In a previous study, butyrate significantly reduced NO production in LPS-stimulated RAW cells, and the inhibition was reversed by the removal of butyrate (46). Another study reported from the same group showed that butyrate increased NO production in the murine vascular endothelial cell line (END-D) in response to LPS or IFN-γ (47). Recently, NaB was reported to markedly suppress macrophage-driven inflammation in a non-alcoholic steatohepatitis model. It suppressed LPS-induced proinflammatory gene expression (TNF-α, IL-6) and their secretion (48). This may indicate that the anti-inflammatory effect of NaB and the combination may play a role in their antiproliferative effects against the AGS cells.

### 3.5 Flow cytometric analyses of apoptotic profiles of mono and combination therapies

In this assay, Annexin V and 7-AAD were used to detect apoptosis and necrosis, respectively using flow cytometry. Annexin V binds phosphatidylserine (at the outer surface of cells during apoptosis) and is used to detect apoptosis, while 7-AAD (with a high affinity for guanine-cytosine residues) intercalates the double-stranded DNA indicating necrosis (49).

**Figure 3:**
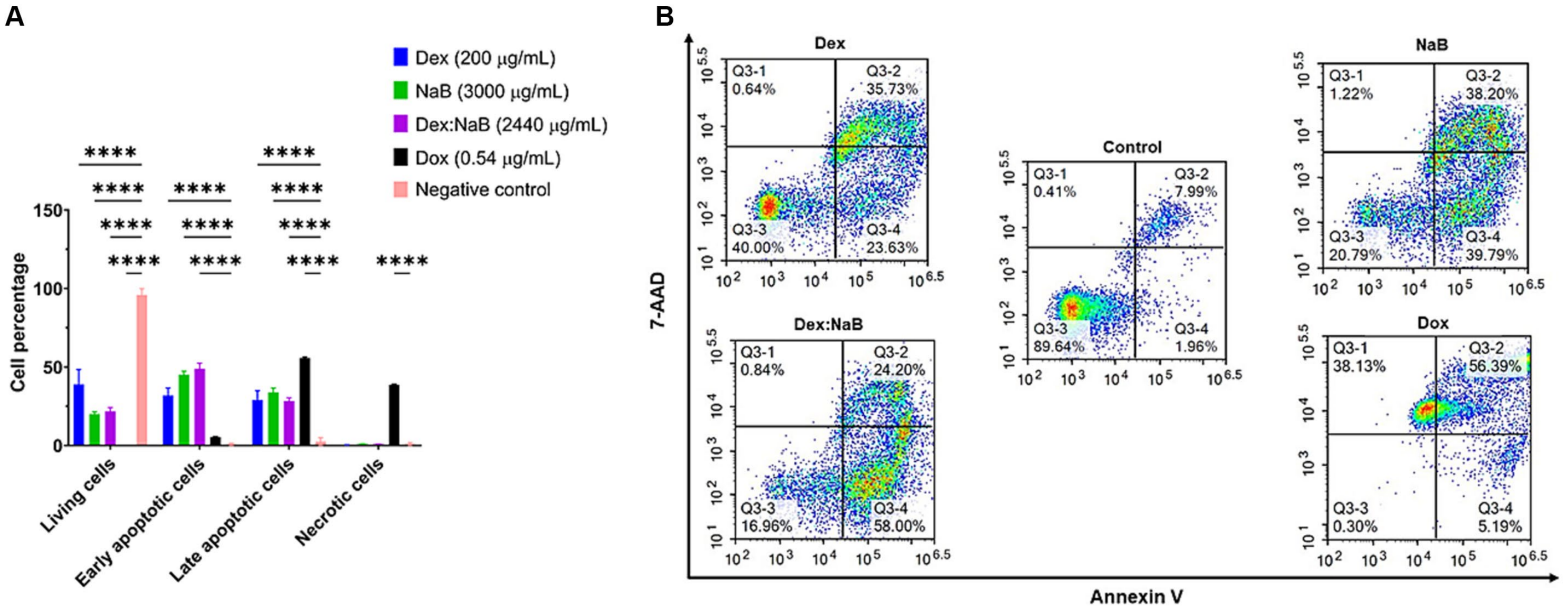
Flow cytometric assessment of the apoptotic profiles of the AGS gastric cancer cells after 24 h of treatment. **(A)** The live, early apoptotic, late apoptotic, and necrotic cell percentages after 24 h treatment with NaB (3,000 μg/mL), Dox (0.54 μg/mL), Dex (200 μg/mL), Dex:NaB (40 μg/mL and 2,400 μg/mL, respectively) and control (*n* = 4). *indicates 0.01 < value of *p* <0.05; **indicates *p* < 0.01; ***indicates *p* < 0.001; ****indicates *p* < 0.0001 compared to the negative control. **(B)** Represented are the density plots of each drug treatment that is most representative of the average data from the flow cytometric analyses, with Q3-1 = necrotic cells, Q3-2 = late-stage apoptotic cells, Q3-3 = live cells, and Q3-4 = early stage apoptotic cells.

After 24 h of treatment, significant distinctions in the populations of viable, early apoptotic, and late apoptotic cells were evident in both the individual and combined treatments compared to the control group (Figure 3). Dex (200 μg/mL) treatment resulted in a notable proportion of living cells (38.95%), with a moderate percentage of early apoptotic cells (31.86%) and late apoptotic cells (28.84%), and a very low proportion of necrotic cells (0.35%). NaB treatment (3,000 μg/mL) demonstrated a substantial increase in early apoptotic (45.20%) and late apoptotic cells (33.83%) and a moderate proportion of living cells (20.03%). The synergistic combination of Dex and NaB (Dex: NaB at 40: 2400 μg/mL, respectively) displayed a significant reduction in living cells (21.75%; *p* < 0.0001) and an increase in early apoptotic cells (48.97%; *p* < 0.0001) and comparable levels in late apoptotic cells (28.41%; *p* < 0.0001) to the vehicle control and the mono treatments. Necrotic cells remained at a relatively low level in the Dex-NaB combination group (Figure 3). The positive control Dox (0.54 μg/mL) showed a very low percentage of living cells (0.23%), a moderate increase in early apoptotic cells (5.38%), a substantial percentage of late apoptotic cells (55.75%), and a significant proportion of necrotic cells (38.66%; Figure 3). The synergistic Dex-NaB combination demonstrated a notable increase in early apoptotic cells compared to the monotreatments, suggesting a potential synergy in inducing apoptosis. These findings highlighted the potential of the Dex:NaB combination in promoting apoptotic pathways and warrant further investigation into its therapeutic implications.

### 3.6 Proteomics study of the AGS cells treated with the synergistic combination vs. mono treatments

The bottom-up label-free quantification proteomics analysis using NanoUPLC-qTOF-MS was performed as per the protocol from a recent study from our group to understand the primary difference in the expression of key biomarkers (related to apoptotic and cancer pathogenesis) after treatment with the most potent postbiotic- NaB, its most synergistic combination with Dex (2:8), Dex alone and the vehicle control (19). The expressed proteins in NaB only, Dex only and 2:8 Dex-NaB combination treated AGS cells were analyzed in pairwise comparisons to the control group to trace the difference in the proteome-wide dysregulated expressions that may be associated with the cytotoxic effects of the combination. The proteins that exhibited differential expression in the treated AGS cells, in comparison to the control group, were chosen based on significance levels, with an adjusted value of p of ≤0.01 alongside an absolute FC ≥ 2 (absolute log2 FC ≥ 1) cutoffs.

The proteins that demonstrated statistical significance as a result of the analysis were subsequently classified according to whether they were upregulated or downregulated in response to the treatments. In order to elucidate the molecular pathways that might be associated with the quantified proteins, different online tools were used to identify the potential pathways activated by the dysregulated proteins in pairwise comparisons, including Reactome (version 83), UniProt (release 2022_04), g:Profiler (Ensembl version 107, Ensembl Genomes version 54, Wormbase ParaSite version 17), and STRING (version 12.0).

#### 3.6.1 Protein identification and quantification in the AGS cells after the treatment with NaB

Several studies have investigated the effect of SCFA butyrate against different types of cancer, however, few have focused on GC (50–52). In the current study, NaB was determined to be the most effective gut microbial metabolite against the AGS gastric cancer cells in inducing apoptosis and ROS production. Table 4 displays the most significant differentially dysregulated genes along with the possible pathways and mechanisms of action upon treatment with NaB, Dex and Dex-NaB (2:8) combination. NaB significantly regulated the activity of signal transduction, metabolism, chromatin organization, cell adhesion, and keratinization. The proteomic analysis of the NaB group revealed a subset of downregulated proteins encoding genes, including *TNS4, UBE2C, DSG2, COL17A1*, and *CCP110* and a subset of overregulated proteins encoding genes, including *KAT6A, KAT6B, IGSF9B* and *KRT17-19.* Tensin-4 (*TNS4*) downregulation showed the potential to exhibit activity independently and in combination with the expression of other genes following NaB treatment (Table 4). *TNS4* is an emerging oncogene essential for cancer cell survival and proliferation (53). It was previously reported that *TNS4* directly interacts with phosphorylated MET tyrosine kinase, which positively influences cell survival, proliferation, and migration (53). *TNS4* expression is upregulated by epidermal growth factor (EGF), and elevated *TNS4* mediates EGF-induced cell migration (54). The combined downregulation of *TNS4*, along with five potentially upregulated (*KRT8, KRT17, KRT18, MT-CO2, BAIAP2*) genes and five other downregulated proteins (CRKL, DSG2, COL17A1, RBM3, LPP) indicates a potential initiation of signaling via EGFR1 (Table 4). Ubiquitin-conjugating enzyme E2C (*UBE2C*) is a potential oncogene that plays a crucial role in governing cell cycle advancement and contributes to the development of various cancers (55). *UBE2C* receives ubiquitin from the E1 complex and facilitates its covalent attachment to other proteins (known as ubiquitination) (56). Knockdown of *UBE2C* led to the obstruction of G_2_/M phase transition in intestinal-type GC cells (derived from clinical 1,868 GC cases) (56). In the current study, the combined effect of *UBE2C* with four other dysregulated genes indicates a potential dysregulation of the mitosis leading to cancer cell death in the AGS gastric adenocarcinoma cells (Table 4).

**Table 4:** The upregulation (red) and downregulation (green) of the most prolific proteins and genes (*p* < 0.02) by NaB, Dex, and their combination in the AGS gastric adenocarcinoma cell line, including the associated molecular pathways and mechanisms of action induced by the expression of these proteins and genes.

Treatment	FC	*Gene ID*	Molecular pathway	Mechanism of action
NaB	***−11.6***	***TNS4***	Signal Transduction	Signaling by MET Promotes EGF-induced cell migration.
	***−14.1***	***UBE2C***	Metabolism	Metabolism of protein (gastric cancer network).
	***2*** ***2.1***	***KAT6A*** ***KAT6B***	Chromatin modifying enzymes	HATs acetylate histones – chromatin organization
	***16.3***	***IGSF9B***	Cell adhesion	Trans-activated by p53 and inhibits cancer metastasis by modulating FAK/AKT signaling pathway.
	***3.6*** ***2.4*** ***3.0*** ***3.6*** ***2.2*** ***−2.6*** ***−11.6*** ***−7.6*** ***−3.3*** ***−2.5*** ***−2.2***	***KRT8*** ***KRT17*** ***KRT18*** ***MT-CO2*** ***BAIAP2*** ***CRKL*** ***TNS4*** ***DSG2*** ***COL17A1*** ***RBM3*** ***LPP***	Signal Transduction	EGFR1 pathway
	***2.4*** ***3.0*** ***3.9*** ***2.3*** ***2.0*** ***2.0*** ***−7.6***	***KRT17*** ***KRT18*** ***KRT19*** ***KRT6B*** ***KRT8*** ***KRT80*** ***DSG2***	Keratinization	Modulates the function of TNF-alpha
	***2.4*** ***2.2*** ***2.8*** ***−11.6***	***FGFR4*** ***BAIAP2*** ***CTNNA1*** ***TNS4***	Signal Transduction	Signaling by receptor tyrosine kinase
				VEGFA-VEGFR2 Pathway
	***2.8*** ***−3.3*** ***−2.8*** ***−2.7***	***CTNNA1*** ***COL17A1*** ***CLDN7*** ***PARVA***	Cell–Cell communication	Cell junction organization
	***2.8*** ***3.0*** ***3.2***	***VDAC1*** ***VDAC2*** ***VDAC3***	Transport of small molecules.	Mitochondrial calcium ion transport

	***2.1*** ***2.2*** ***−14.1*** ***−4.3*** ***−2.7***	***CKAP5*** ***CEP57*** ***UBE2C*** ***CCP110*** ***NUP50***	Cell cycle	M Phase
	***89.5*** ***2.8*** ***−2.1*** ***−2.2***	***PLEKHA7*** ***CTNNA1*** ***DIAPH1*** ***LPP***	Signal Transduction	Stabilization and expansion of the E-cadherin adherens junction
	***2.1*** ***2.8*** ***2.2*** ***−2.1*** ***−4.2*** ***−2.2***	***CKAP5*** ***CTNNA1*** ***BAIAP2*** ***DIAPH1*** ***RTKN*** ***KIF5B***	Signal transduction	RHO GTPase Effectors
	***2.8*** ***3.5***	***VDAC1*** ***TOMM40***	Selective autophagy	Mitophagy
	***35.4***	***ARL3***	Vesicle-medicated transport	Membrane trafficking
	***2.1*** ***2.2*** ***−4.3***	***CKAP5*** ***CEP57*** ***CCP110***	Cell cycle	G_2_-G_2_/M Phase. Loss of proteins required for interphase microtubule organization from the centrosome
	***35.4*** ***3.4*** ***2.2*** ***2.1*** ***−4.3***	***ARL3*** ***IFT22*** ***CEP57*** ***CKAP5*** ***CCP110***	Organelle biogenesis and maintenance	Cilium Assembly
	***34.1*** ***2.6*** ***3.6*** ***2.8*** ***4.3*** ***−2.6*** ***−14.1*** ***−2.7***	***TXNIP*** ***HBA2*** ***MT-CO2*** ***HSPA2*** ***MT2A*** ***SOD1*** ***UBE2C*** ***NUP50***	Cellular responses to external stimuli	Cellular response to stress
***5.1*** ***−4.3*** ***−2.2***	***MGST3*** ***RRM1*** ***SRM***	Glutathione metabolism	Metabolism
Dex	***2.9*** ***2.0*** ***2.8***	*CYP51A1* ***HMGCS1*** ***FDFT1***	Cholesterol Biosynthesis Pathway	Activates cholesterol biosynthesis pathway.
	***2.5*** ***2.2*** ***5.9*** ***−2.2***	***VDAC2*** ***HMOX1*** ***VDAC3*** ***TFRC***	Ferroptosis	Iron-dependent cell death.
	***2.9*** ***2.4*** ***3.1*** ***2.2*** ***2.2*** ***−2.2*** ***−3.0*** ***−2.7*** ***−2.2***	***KRT18*** ***KRT5*** ***KRT8*** ***CTTN*** ***RALB*** ***RBM3*** ***CRKL*** ***COL17A1*** ***TFRC***	Signal transduction	EFGR pathway
	***−21.4***	***UBE2C***	Metabolism.	Metabolism of protein (gastric cancer network).
	***−4.5***	***RACK1***	Signal transduction	Protein C kinase
Dex:NaB 2:8	***−12.1***	***TNS4***	Signal Transduction	Signaling by MET Promotes EGF-induced cell migration.
	***2.5***	***KAT6B***	Chromatin modifying enzymes	HATs acetylate histones – chromatin organization
	***2.3***	***IGSF9B***	Cell adhesion	Trans-activated by p53 and inhibits cancer metastasis by modulating FAK/AKT signaling pathway.
	***3.8***	***ATP5PF***	Oxidative phosphorylation	Via modulation of citric acid (TCA) cycle and respiratory electron transport
	***2.3***	***NDUFAB1***		

FC, Signed maximum fold change calculated by Progenesis QIP where the negative sign denotes downregulation.

Chromatin organization-associated proteins were upregulated, such as K acetyltransferase 6A and its paralog 6B, encoded by *KAT6A* and *KAT6B* in the NaB-treated cells. These histone acetyltransferase enzymes catalyze the acetylation of histones on specific lysine residues (57). They also interacted with p53 (probably by acetylation) to induce p21 expression and cell-cycle arrest in the MCF7 breast adenocarcinoma cells (58, 59). *KAT6A* and *KAT6B* have been reported to stimulate the expression of Brahma (BRM), an anticancer gene often inactivated in various tumor types (60). Additionally, *KAT6B* undergoes genomic loss in small-cell lung cancer, classifying it as a tumor suppressor histone acetyltransferase (61). The expression of *KAT6B* was found to be significantly lower in hepatocellular carcinoma cells previously (62). Furthermore, there are reports of interactions between KAT6B and ING5, members of the ING family of tumor suppressors (63). Therefore, the upregulation of these genes may be implicated in the anticancer activity of NaB. However, other contradictory studies have demonstrated that inhibitors of histone acetyltransferases KAT6A/B induce senescence and halt tumor growth (64). The results of the current study align with the reported efficacy of butyrate as a nonspecific histone deacetylase (HDAC) inhibitor, inducing p21 expression and consequently leading to cell differentiation and apoptosis in cancer cells (65).

Immunoglobulin superfamily (IgSF) is a cell adhesion molecule that mediates cell–cell interaction and transduces intracellular signals (66). *IGSF9B* is a member of IGSF that was reported to be downregulated in colorectal familial adenomatous polyposis, and melanoma while upregulated in ovarian cancer, endometrial cancer, and gallbladder cancer (67). *IGSF9* was reported to be activated by tumor suppressor gene p53 and inhibits metastasis of breast cancer by modulating the FAK/AKT signaling pathway. Interestingly, NaB treatment led to the upregulation of IGSF9B, which could be one of the potential mechanisms contributing to the antiproliferative effects of NaB compared to the control.

**Figure 4:**
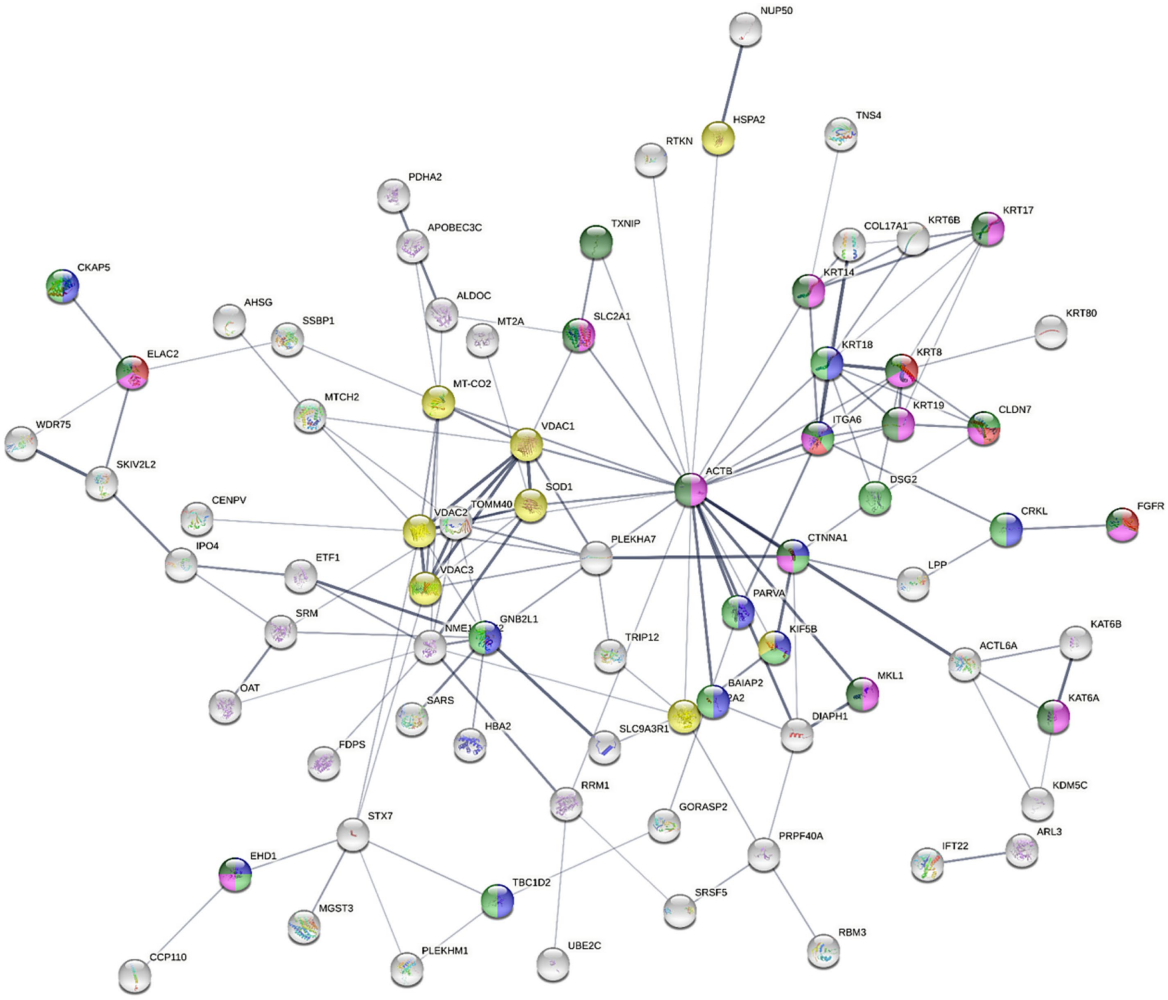
The STRING network illustrates the interconnectedness of dysregulated genes in the AGS gastric adenocarcinoma cells following NaB treatment for 24 h against control cells (absolute FC ≥ 2 and adjusted *p* value≤0.05). The connecting lines visualize the interactions between these genes, with thicker lines representing stronger associations and thinner lines indicating weaker connections. The network was constructed using medium confidence as the minimum required interaction score (0.4). Text mining, experiments, databases, co-expression, neighborhood, gene fusion and co-occurrence as active interaction sources. The full STRING network was applied (the edges indicate both functional and physical protein associations), where disconnected nodes were hidden. Node colored to indicate specific ontology [Red; cell adhesion molecule binding (molecular function; MF), blue; Cadherin binding (MF), green; Cancer Disease-gene associations (DISEASES)], Turquise; Disease of cellular proliferation [Disease-gene associations (DISEASES) and yellow; prion disease (KEGG pathways)].

The upregulation of *KRT17, KRT18, KRT19, KRT6B, KRT8,* and *KRT80,* in combination with the downregulation of *DSG2* in the NaB-treated cells indicated a potential modulation of keratinization. *KRT17* was reported to suppress cell proliferation in pancreatic cancer patients. A previous report showed that human embryonic kidney (HEK) cells treated with butyrate expressed higher levels of both markers of differentiation, including Keratin-1 (*KRT1*) and *KRT10* (68). The combined upregulation of *PLEKHA7* and *CTNNA1* and the downregulation of *DIAPH1* and *LPP* initiated a possible dysregulation of E-cadherin and E-cadherin-based activity required for collective invasion and migration. The association between potential genes dysregulated by NaB treatment is illustrated in Figure 4.

#### 3.6.2 Protein identification and quantification in the AGS cells after the treatment with Dex

**Figure 5:**
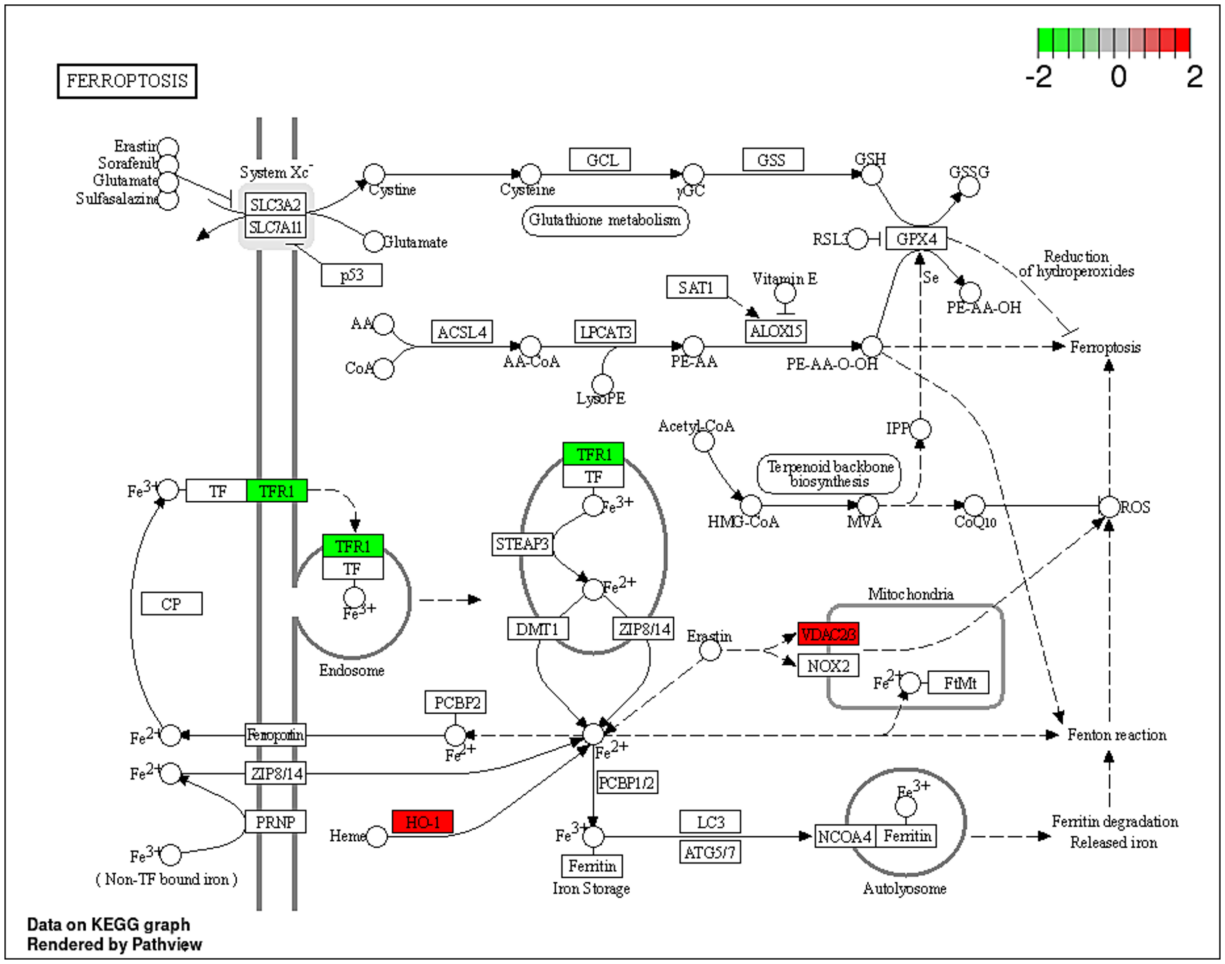
A schematic representation of the ferroptosis pathway. Proteins indicated in red were significantly upregulated (*VDAC2*, *VDAC3* and *HO*-1; *HMOX1*) within the ferroptosis pathway, and proteins in green were downregulated (TRF1) in the Dex-treated AGS gastric adenocarcinoma cells. https://www.bioinformatics.com.cn/srplot was used to generate the figure.

Dex is a glucocorticoid with anti-inflammatory and immunosuppressive activities (69). Dex treatment was reported to improve the sensitivity to chemotherapies by inducing cell death by suppressing cell survival pathways mediated by the glucocorticoid receptor (GR) in hepatocellular carcinoma HepG2 and the AsPC-1 pancreatic ductal adenocarcinoma cells (70). Unsurprisingly, in the current study, Dex treatment resulted in dysregulated expression of proteins involved in cholesterol biosynthesis, namely *CYP51A1*, *HMGCS1* and *FDFT1* genes. Additionally, the upregulation of *VDAC2*, *VDAC3,* and *HMOX1* in combination with the downregulation of *TFRC* indicated potential induction of ferroptosis (Figure 5). Ferroptosis is a programmed cell death distinguished by the iron-dependent accumulation of lipid hydroperoxides (71). The voltage-dependent anion channel (VDAC; Figure 5) is a pore situated on the outer membrane of the mitochondrion, facilitating the passage of various ions and metabolites between the cytosol and the mitochondrion (71). VDAC regulates different cellular processes, including ion homeostasis and apoptosis (71). Erastin is a small molecule that initiates ferroptosis via activation of VDAC (72). Erastin treatment also resulted in upregulation of the expression of heme oxygenase 1 (HMOX1) (73) which is, a pro-ferroptosis enzyme, that catalyzes the breakdown of heme into carbon monoxide, ferrous iron, and biliverdin (74). Ferrous iron was reported to accelerate erastin-triggered ferroptosis cell death (74).

Transferrin receptor protein 1 (*TRF1*; Figure 5) is a membrane glycoprotein that mediates cellular uptake of iron from a plasma glycoprotein, transferrin (74). TRF1 was overexpressed in several cancer types, including GC, renal cell carcinoma and glioblastoma multiforme (75). A previous study showed that long-term use of Dex reduced liver iron concentration and downregulated hepatic *TFR1* in Rats (76). The results of the current study align with a previous investigation, which indicated that Dex sensitizes ferroptosis through glutathione depletion and the induction of dipeptidase-1 expression by the glucocorticoid receptor (73). However, the current study provides an in-depth explanation of the potential mechanism of Dex-dependent ferroptosis, and the dysregulated proteins associated with the process.

Additionally, the upregulation of *KRT5*, *KRT8*, *KRT18*, *CTTN* and *RALB,* in combination with the downregulation of *RBM3*, *CRKL*, *COL17A1* and *TFRC* indicated a potential signal transduction modulation. Dex treatment (Table 4) potentially downregulated the receptor of activated protein C kinase 1 (*RACK1*) gene expression. The overexpression of *RACK1* was associated with cancer progression and poor prognosis (77). RACK1 upregulation was reported to promote cancer progression via the NF-κB pathway (by increasing the M2/M1 macrophage ratio) in oral squamous cell carcinoma previously (78).

#### 3.6.3 Protein identification and quantification in the AGS cells after the treatment with Dex-NaB (2:8) combination

**Figure 6:**
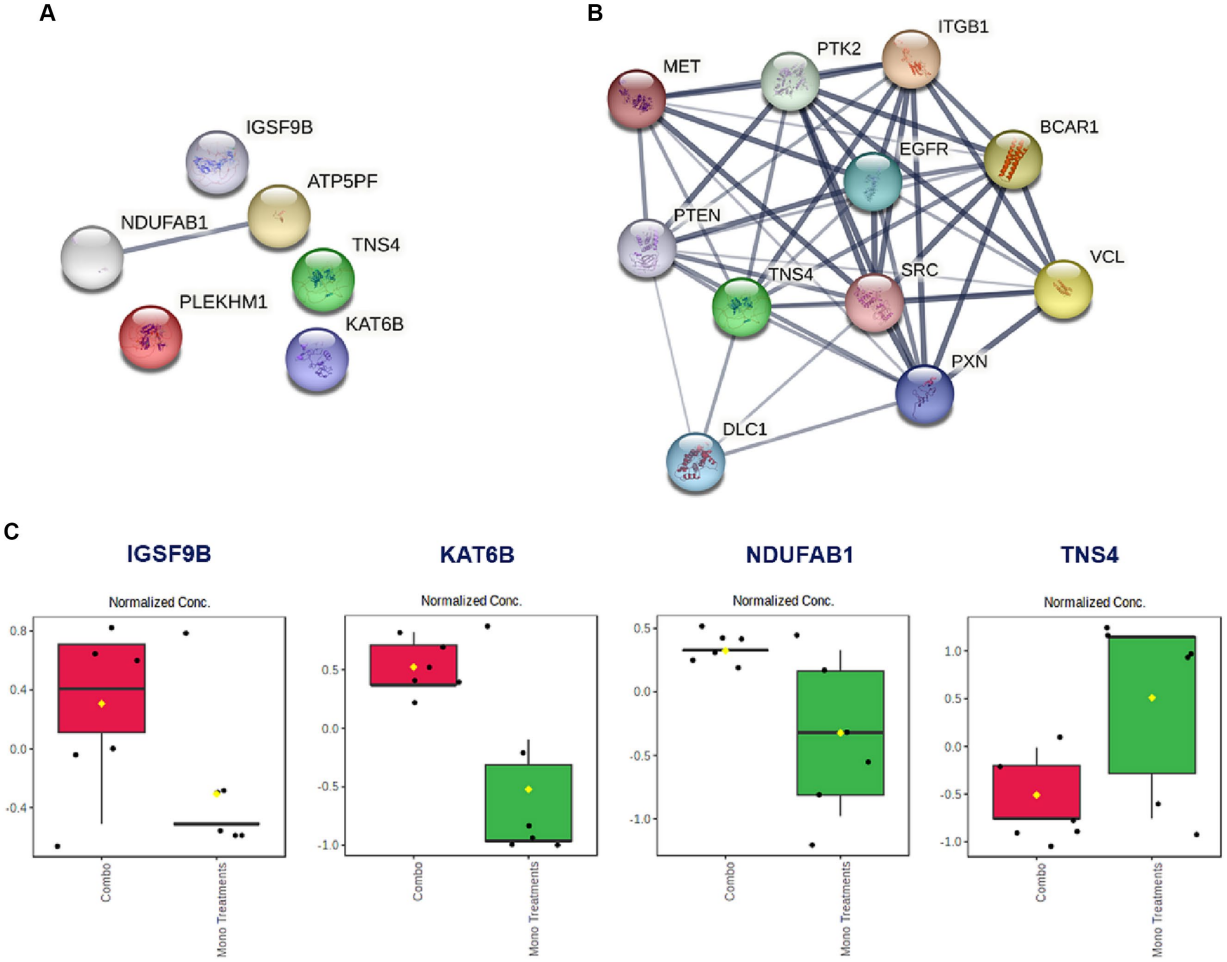
**(A)** The STRING network illustrates the genes that exhibit altered expression levels in the AGS gastric adenocarcinoma cells following the combination treatment (2:8 of Dex and NaB) for 24 h. **(B)** STRING network to display the *TNS* gene and the top 10 interconnected genes. The connecting lines visualize the interactions between these genes, with thicker lines representing stronger associations and thinner lines indicating weaker connections. **(C)** Normalized concentration of the expression of selected proteins in the combination therapy compared to the monotherapies.

Based on the promising antiproliferative activity of the Dex-NaB (2:8) combination against the AGS gastric adenocarcinoma cells, we studied the proteins associated with this activity and compared the proteomic identification between mono and combination therapies. Many essential proteins were significantly upregulated compared to the untreated control, including histone acetyltransferase (KAT6B), ATP synthase-coupling factor 6 (ATP5PF), acyl carrier protein (NDUFAB1), and immunoglobulin superfamily member 9B (IGSF9B). Additionally, certain proteins were downregulated, such as tensin 4 (TNS4) and pleckstrin homology domain-containing family M (PLEKHM1), in the combination group compared to mono treatments (Table 4; Figure 6). A list of dysregulated genes by the combination treatment is illustrated in Figure 6.

Interestingly, the combination therapy induced the downregulation of the oncogene *TNS4* significantly more than the butyrate monotreatment (fold change = −12.1), which may be attributed to the synergistic combination with Dex. In line with our finding, a previous report showed that the tumor suppressor gene phosphatase and tensin homolog (*PTEN*) was upregulated upon Dex treatment in the A549 lung cancer cells (79) (Figure 6B). Additionally, Dex was reported to inhibit hepatocyte growth factor (HGF) dose-dependently through its intracellular receptor in rat hepatocytes (80). Both *PTEN* and *HGF* (via MET) directly interact with *TNS4* expression in cells and could be implicated in the synergistic inhibition of *TNS4* in the combination group compared to mono treatments (81, 82).

NaB treatment resulted in the upregulation of the tumor suppressor gene KAT6B (Table 4). Surprisingly, the Dex-NaB (2:8) combination exhibited higher upregulation of KAT6B compared to monotherapies (Figure 6C). Dex at low concentrations (10^−12^ and 10^−10^ M) has been documented to inhibit histone acetylation, while at higher concentrations (10^−8^ and 10^−6^ M), it can induce histone acetylation at specific target lysine residues in the A549 lung cancer cells (83). This may result in more pronounced upregulation in the combination group compared to monotherapies.

## 4 Conclusion

All three SCFA salts MgA, NaP and NaB exhibited antiproliferative activity against the AGS gastric adenocarcinoma cells with NaB displaying the greatest inhibitory effects (*p* < 0.05). The study evaluated potential synergistic interactions between Dex and NaB, demonstrating strong synergy at a 2:8 ratio (40 μg/mL Dex + 2,400 μg/mL NaB) against the AGS cells, as indicated by CI values <1, surpassing the effects of individual treatments. Dex and NaB individually and in combination (2:8) showed significant changes in cell populations, with the combination indicating a shift toward apoptosis, as evidenced by reduced living cells and increased early apoptotic cells. MgA and NaP consistently decreased ROS production, while NaB showed a notable increase, suggesting pro-oxidative properties. Dex exhibited antioxidative effects, and in the combination of NaB and Dex potentially counteracted the pro-oxidative effects of NaB highlighting an interaction. Several key proteins belonging to the biological processes, cellular components, and molecular function categories in the AGS cells were dysregulated upon treatment with the synergistic combination compared to the mono treatments. Notably, oncogene *TNS4* was significantly downregulated in the combination group in comparison to the monotherapies. These findings indicate the potential implementation of NaB as adjuvant therapy with Dex in GC. Further investigations into the underlying mechanisms of this combination effect would contribute to a more comprehensive understanding of its significance in the context of GC.

## Data availability statement

The datasets presented in this study can be found in online repositories. The names of the repository/repositories and accession number(s) can be found in the article/Supplementary material.

## Ethics statement

Ethical approval was not required for the studies on humans and animals in accordance with the local legislation and institutional requirements because only commercially available established cell lines were used.

## Author contributions

RE: Conceptualization, Data curation, Formal analysis, Investigation, Methodology, Project administration, Visualization, Writing – original draft, Writing – review & editing. MA: Data curation, Investigation, Methodology, Writing – review & editing. DC: Writing – review & editing. MF: Data curation, Visualization, Writing – original draft. C-GL: Writing – review & editing. DB: Conceptualization, Funding acquisition, Methodology, Project administration, Resources, Supervision, Visualization, Writing – original draft, Writing – review & editing.
